# Altered Expression of Circadian Clock Genes in Patients with Atrial Fibrillation Is Associated with Atrial High-Rate Episodes and Left Atrial Remodeling

**DOI:** 10.3390/diagnostics11010090

**Published:** 2021-01-07

**Authors:** Yung-Lung Chen, Jiin-Haur Chuang, Hui-Ting Wang, Huang-Chung Chen, Wen-Hao Liu, Ming-Yu Yang

**Affiliations:** 1 Section of Cardiology, Department of Internal Medicine, Kaohsiung Chang Gung Memorial Hospital, Kaohsiung 833, Taiwan; feymanchen@gmail.com (Y.-L.C.); chc3@cgmh.org.tw (H.-C.C.); wenhao@cgmh.org.tw (W.-H.L.); 2 Graduate Institute of Clinical Medical Sciences, College of Medicine, Chang Gung University, Taoyuan 333, Taiwan; jhchuang@cloud.cgmh.org.tw; 3 Division of Pediatric Surgery, Department of Surgery, Kaohsiung Chang Gung Memorial Hospital, Kaohsiung 833, Taiwan; 4 Emergency Department, Kaohsiung Chang Gung Memorial Hospital, Kaohsiung 833, Taiwan; gardinea1983@gmail.com; 5 Department of Otolaryngology, Kaohsiung Chang Gung Memorial Hospital, Kaohsiung 833, Taiwan

**Keywords:** atrial fibrillation, circadian rhythm, circadian clock genes, burden of atrial high-rate episodes, cardiac remodeling

## Abstract

A prominent circadian variation is present in atrial fibrillation (AF) attacks that may be related to the expression of circadian clock genes. Little is known about the expression of circadian clock genes in AF. We prospectively enrolled 73 patients who had received pacemaker implantation, in order to define the burden of atrial high-rate episodes (AHREs) accurately. AF was diagnosed clinically in 43 (59%) patients (15 with persistent AF and 28 with paroxysmal AF). The expression levels of circadian clock genes of peripheral blood leukocytes were checked. There were more males and patients with a larger left atrial (LA) size and lower expression levels of *BMAL1*, *CRY2*, *NR1D1*, *NR1D2*, *PER2*, *RORA*, *RORC*, and *TIM* genes in persistent AF group than in other groups. There was a significant correlation between higher AHRE burden and larger LA size and between higher AHRE burden and decreased expression of circadian clock genes in patients with AF. LA volume and the expression of *CRY1*, *NR1D1*, and *RORA* are significantly associated with AHRE burden. However, the underlying mechanism needs to be elucidated in further studies.

## 1. Introduction

Atrial fibrillation (AF) is one of the most common cardiovascular diseases in patients, and it increases the risk of embolic stroke, heart failure, and mortality compared to patients without AF [1]. According to a previous study, a prominent circadian variation is present in AF episodes in patients with paroxysmal AF [2]. Circadian clock genes are defined as genes whose protein products are necessary components for the generation and regulation of circadian rhythms. At least 14 circadian clock genes have been found in humans; these are *BMAL1*, *CK1ε*, *CLOCK*, *CRY1*, *CRY2*, *NRID1*, *NR1D2*, *PER1*, *PER2*, *PER3*, *RORA*, *RORB*, *RORC*, and *TIM*. A major feature of circadian rhythm is that the mRNA and protein of the circadian clock genes and the mRNA and protein of the genes regulated by circadian rhythm exhibit distinct daily day–night fluctuation cycles [3,4]. Some cardiovascular or metabolic diseases, such as hypertension and diabetes, have been shown to be involved in the regulation of physiological clock genes [5]. The onset of acute myocardial infarction and arrhythmias is also regulated by circadian clock genes [6]. A recent study using the Taiwan National Health Insurance Research Database showed that the incidence of AF development was significantly higher in insomnia cases than in the comparison cohort [7], although the underlying mechanism is still unknown. Those genes that control the ion channel, gap junctions and atrial fibrosis may also be regulated by circadian clock genes (which are known as circadian controlled genes) and are associated with the pathophysiologic mechanism of AF [8,9,10].

We chose patients who received permanent pacemaker (PPM) implantation due to sick sinus syndrome to include in this study, so as to have an accurate diagnosis of AF type and document the burden of atrial high-rate episodes (AHREs). As such, this study investigated the expression of circadian clock genes and AHRE burden, with the goal of elucidating the association of circadian clock genes with AF.

## 2. Materials and Methods

### 2.1. Patient Enrollment and Sample Management

**Figure 1:**
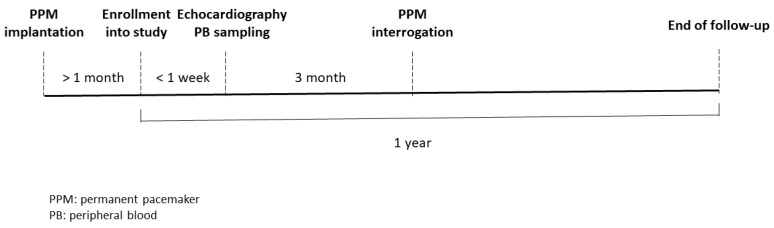
Flow chart of study design.

Patients who were diagnosed with sick sinus syndrome and had received permanent pacemaker implantation at our institute were enrolled into this study from August 2018 through December 2019. Patients with autoimmune disease, malignancy, and chronic inflammation status were excluded from the study. We enrolled patients into this study at least one month after the implantation of the pacemaker to prevent the inflammatory status of post-pacemaker implantation may influence the genes expression. The peripheral blood (PB) sample and echocardiographic study were performed at the same day within one week after patients being enrolled into this study. The flow chart of study design was shown in Figure 1. The patients’ PB samples were collected between 8:00 a.m. and 9:00 a.m., and total leukocytes from the PB of patients were used for analysis of circadian clock genes expression. The patient’s clinical characteristics, including age, sex, comorbidities such as a history of AF, and echocardiographic findings, were analyzed. The definition of heart failure in baseline characteristics was according to diagnosis of previous hospitalization, which include heart failure with reduced and preserved ejection fraction.

The patients enrolled in this study did not experience shift work or jet lag one month before the collection of PB. They were asked to have breakfast between 6:00 and 8:00, lunch between 11:00 and 13:00, and dinner between 17:00 and 19:00 during the one month before collection of PB; they were also asked to go to sleep before 23:00 and wake up at around 7:00 during the same period.

### 2.2. Definition of AF Type and Atrial High-Rate Episodes and Measurement of Left Atrial (LA) Diameter and LA Volume

Electrocardiography (ECG) and Holter tests of studied patients were reviewed in detail by 2 electrophysiologic (EP) doctors. PPM interrogation and intracardiac electrograms evaluation, also by 2 EP doctors, were performed every 3 months to confirm the occurrence of AF and the duration of atrial high-rate episodes (AHREs). AF type was classified as paroxysmal if there was AF lasting more than 30 s, as measured by ECG or Holter test, with sinus rhythm documented by ECG within one week of the AF episodes, Holter test, or intracardiac electrograms stored in the device recorder. Persistent AF was defined as the longest AF duration of more than one week without any sinus rhythm documented by ECG, Holter test, or intracardiac electrograms stored in the device. AHREs were detected by the device automatically and defined as an episode of fast atrial rate ≥ 180 beats per minute lasting at least 5 min, which included atrial tachycardia, atrial flutter, and AF, according to 2016 European Society of Cardiology guidelines [11]. The stored intracardiac electrograms were confirmed by the EP doctors to exclude artifacts and far-field signals. The measurement of left atrial (LA) diameter is conducted by two-dimensional measure, which is perpendicular to the long axis of the LA posterior wall, inner edge to inner edge, at the level of the aortic sinuses. LA volume was measured using the area-length method using the apical 4-chamber and apical 2-chamber views at the end-ventricular systole, when the LA is at its maximal size [12]. The patients were followed for one year. This study was approved by the Institutional Review Board of Chang Gung Memorial Hospital (IRB number: 201702224B0). This study conformed to the guidelines set forth by the Declaration of Helsinki. Written informed consent was obtained from the participants before starting the study.

### 2.3. Real-Time Quantitative Reverse Transcriptase-Polymerase Chain Reaction Analysis (qRT-PCR) of Circadian Clock Genes

Isolation of total PB leukocytes, RNA extraction, cDNA synthesis, and qRT-PCR were performed as previously described [13,14]. Briefly, the 2 μg RNA input for cDNA synthesis was determined by spectrophotometric OD_260_ measurement, and cDNA was generated with a High Capacity cDNA Reverse Transcription Kit (Applied Biosystems, Foster City), following the manufacturer’s protocol. The expression of the 14 circadian clock genes was analyzed using the TaqMan system. TaqMan Gene Expression Assays for *NR1D1*, *NR1D2*, *RORA*, *RORB*, and *RORC* were purchased from Applied Biosystems (Waltham, MA, USA). The specific forward and reverse primers and TaqMan probe of the other 9 genes were designed by Applied Biosystems, and the TaqMan MGB probes were synthesized and labeled with appropriate fluorescent dyes by Applied Biosystems, as previously described [13,14]. Expression of the human housekeeping gene, *ACTB* (β-actin), was used for normalizing circadian clock genes in real-time qRT-PCR. All reactions were carried out in a 10 μL final volume containing 25 ng cDNA (as total input RNA), 400 nM of each primer, 200 nM of probe, and 5 μL of 2X TaqMan Universal PCR Master Mix (Applied Biosystems). Real-time qRT-PCR was performed in an ABI 7500 Fast Real-Time System (Applied Biosystems), and the PCR cycling parameters were set as follows: A temperature of 95 °C for 10 min, followed by 40 cycles each of PCR reactions at 95 °C for 20 s and at 60 °C for 1 min. The expression levels of the circadian clock genes were normalized to the internal control *ACTB* to obtain the relative threshold cycle (∆Ct). All reactions were run in duplicate.

### 2.4. Statistical Analysis

The differences in categorical variables between patients with and without AF based on their history before PPM implantation, and also their PPM interrogation data, were analyzed by chi-square or Fisher’s exact test. One-way ANOVA followed by Bonferroni multiple comparison procedure or Kruskal–Wallis test followed by Dunn’s multiple comparison procedure were used to analyze the differences in continuous variables among 3 groups as an appropriate approach. AHRE burden was expressed as the percentage of the total AHRE duration as defined by device automatically during a 3-month period. The differences of expression in the circadian clock genes between AF group and non-AF group using the ∆Ct values were analyzed by Mann–Whitney test. The differences of expression in the circadian clock genes among no AF group, paroxysmal AF group and persistent AF group using the ∆Ct values were analyzed by Kruskal–Wallis test followed by Dunn’s multiple comparison procedure. The folds change in circadian clock genes mRNA expression in patients with and without AF was determined by 2^-∆∆Ct^ calculation. The level of gene expression in patients with persistent AF, paroxysmal AF, or no AF was also calibrated to obtain the folds changed using 2^-∆∆Ct^ calculation. Bivariate correlation analysis was used for variables, including AHRE burden, LA size, and the expression of circadian clock genes (expressed as −∆Ct value). Multiple linear regression model was performed to assess the association between dependent variables, AHRE burden, and independent variables, namely all the genes showing a significant correlation at univariate analysis, sex, age, and left atrial volume. Finally, we stratified the study population according to tertile distribution on the basis of expression levels of genes, which are significantly associated with AHRE burden, and we later analyzed the linear trend of AHRE burden among these subgroups using Jonckheere-Terpstra test. We used SPSS version 17.0 software (SPSS, Chicago, IL, USA) for all statistical analyses.

## 3. Results

### 3.1. Baseline Characteristics of Patients with No AF, Paroxysmal AF, and Persistent AF

A total of 73 patients were prospectively enrolled. AF was documented in 43 (59%) patients by ECG, 24 h Holter monitoring, and pacemaker interrogation data before enrollment. In all, 15 (21%) patients had persistent AF and 28 (38%) had paroxysmal AF. There were 33 (45%) males, and the average age was 71.5 ± 8.3 years. There was no difference in baseline characteristics, including age, diabetes mellitus, hypertension, stroke, heart failure, vascular disease, chronic kidney disease, anxiety, sedative medications, average heart rate, and echocardiographic parameters, except LA diameter and volume, between patients with and without AF (Table 1). There were more males in the persistent AF group than in the paroxysmal AF and no AF groups (*p* = 0.006). The AHRE burden was higher in the persistent AF group than in the paroxysmal AF and no AF groups (*p* < 0.001). The LA diameter and volume were larger in the persistent AF group than in the paroxysmal AF and no AF groups (both *p* < 0.001).

### 3.2. The Expression of the Circadian Clock Genes in Patients with No AF, Paroxysmal AF and Persistent AF

**Figure 2:**
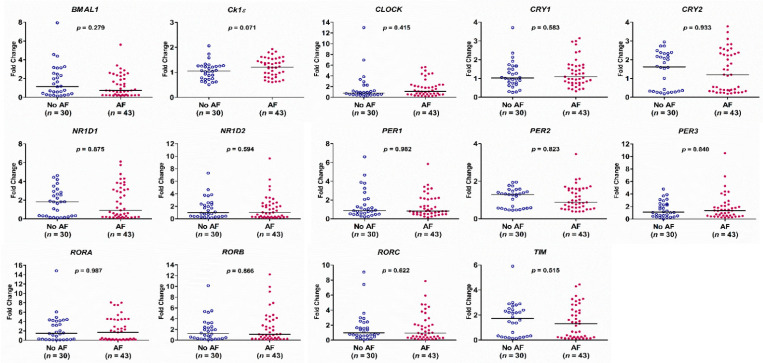
Expression levels of the 14 circadian clock genes in patients with and without atrial fibrillation (AF), as determined by real-time quantitative reverse transcriptase-polymerase chain reaction. The *y*-axis represents the fold change of the gene expression level in patients with AF compared to patients without AF. The *p*-value analyzed by Mann–Whitney test indicates statistical significance as evaluated between patients with AF (*n* = 43) and without AF (*n* = 30) using ∆Ct values.

**Figure 3:**
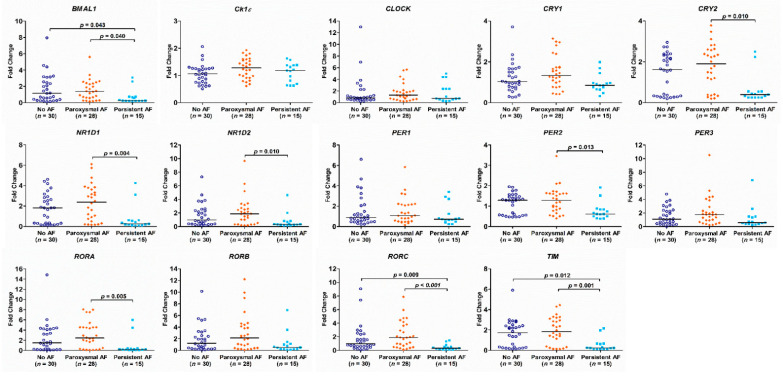
Expression of circadian clock genes in patients with persistent atrial fibrillation (AF), paroxysmal AF or no AF. The *p*-value analyzed by Kruskal–Wallis test followed by Dunn’s multiple comparison procedure indicates statistical significance as evaluated among patients with no AF (*n* = 30), paroxysmal AF (*n* = 28), and persistent AF (*n* = 15) using ∆Ct values.

There was no difference in the expression of the circadian clock genes between the AF group and the no AF group (Figure 2). The expression of *BMAL1*, *CRY2*, *NR1D1*, *NR1D2*, *PER2*, *RORA*, *RORC*, and *TIM* genes in PB leukocytes was significantly different among the three groups (all *p* < 0.05). In detail, the expression of *BMAL1*, *RORC*, and *TIM* genes was significantly lower in patients with persistent AF than in those with paroxysmal AF or no AF (persistent AF vs. paroxysmal AF and persistent AF vs. no AF, all *p* < 0.05). The expression of *BMAL1*, *CRY2*, *NR1D2*, *PER2*, and *RORA* was significantly lower in patients with persistent AF than in those with paroxysmal AF (persistent AF vs. paroxysmal AF, all *p* < 0.05) (Figure 3). Of interest, the expression of these circadian clock genes was not different between patients with paroxysmal AF and no AF, but much lower in the persistent AF group compared with the other two groups.

### 3.3. The Correlation of AHREs, LA Size, and the mRNA Expression of Circadian Clock Genes in Patients with AF

**Figure 4:**
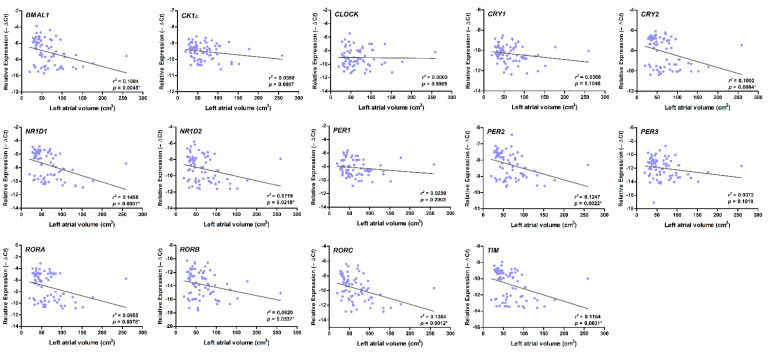
Correlation between the expression of circadian clock genes and left atrial (LA) volume. There was a strong correlation between larger LA diameter and decreased expression of *BMAL1*, *CRY2*, *NR1D1*, *NR1D2*, *PER2*, *RORA*, *RORB*, *RORC*, and *TIM* (all *p* < 0.05), as accessed by bivariate correlation analysis. The *r*- and *p*-values indicate the correlation between the expression of circadian clock genes (expressed as −∆Ct value) and LA volume (expressed as units of cm^3^).

There was also a strong, positive correlation between higher AHRE burden and larger LA diameter and volume, and a strong, positive correlation between higher AHRE burden and decreased mRNA expression of *BMAL1*, *CRY1*, *CRY2*, *NR1D1*, *NR1D2*, *PER2*, *PER3*, *RORA*, *RORB*, *RORC*, and *TIM* in patients with AF (all *p* < 0.05) (Table 2). Larger LA volume was also associated with a decreased expression of *BMAL1*, *CRY2*, *NR1D1*, *NR1D2*, *PER2*, *RORA*, *RORB*, *RORC*, and *TIM* (*p* < 0.05) (Figure 4).

### 3.4. Multiple Linear Regression Model for Assessment of the Association between AHRE Burden and Other Variables

Variables including age, sex, LA volume, and mRNA expression of 11 circadian genes, which were significantly associated with AHRE burden in Table 2, were used to assess the association with AHRE burden. The results showed mRNA expression of *CRY1* [Standardized β coefficient = 0.386, *p* = 0.041], *NR1D1* [Standardized β coefficient = 1.149, *p* = 0.016], *RORA* [β = −1.676, *p* = 0.025], and LA volume [Standardized β coefficient = 0.608, *p* < 0.001] were significantly associated with the AHRE burden (R^2^ = 0.633; *p* < 0.001) (Table 3). The study population was then stratified according to tertile distribution on the basis of expression levels of *CRY1*, *NR1D1*, and *RORA*, and the AHRE burden among these subgroups was analyzed which showed a significant linear trend of these genes expression (*p* < 0.05) (Table 4).

## 4. Discussion

There are several important findings in this study. First, patients with persistent AF had a decreased expression of circadian clock genes compared with the paroxysmal AF and no AF groups. Second, LA volume and the expression of *CRY1*, *NR1D1* and *RORA* were significantly associated with AHRE burden. Third, there was a significant correlation between LA size and decreased expression of circadian clock genes.

Many studies have reported the relationship between AF and sleep disorders, including insomnia and obstructive sleep apnea syndrome [15]. Sleep disorder was associated with a higher AF recurrence rate in patients receiving either medication or ablation therapy [16]. Additionally, sleep disorder will cause alternations in circadian clock genes expression and oscillation [17]. Our previous study found that sleep disorders can affect the performance of physiological clock genes of PB total leukocytes, and cause disease, such as sudden deafness [13]. Since PB is the most accessible source of circadian clock genes from AF patients without a surgical indication, we wanted to evaluate the circadian clock system of AF patients by analyzing circadian clock genes expression in the PB. It would be interesting to know if these patients with AF had an altered circadian clock genes expression and if this altered expression was associated with AF burden. The present study is the first to investigate circadian clock genes expression in patients with AF and found that there was an altered expression of circadian clock genes in different AF types. Since the circadian clock genes expression levels in blood samples have been suggested as appropriate markers for estimating an individual’s circadian rhythm [18], our findings imply that a disrupted circadian rhythm may be associated with the pathophysiologic mechanism of AF.

At present, diurnal changes in the human heartbeat are considered to be related to certain circadian clock gene fragments [19]. In previous studies, patients with an extended *PER3* tandem repeat exhibited an elevated heart rate, and selective deletion of PPAR-γ, a putative activator of *BMAL1*, in the vasculature resulted in diminished heart rate diurnal variations [20,21]. The variability of the heart rate may provide the potential for a transfer to AF [22,23]. Our study also showed that the expression of *BMAL1* and *PER3* was strongly associated with AHRE burden, which conforms to the findings of previous studies. Our study revealed that the expression of *BMAL1*, *CRY2*, *NR1D1*, *NR1D2*, *PER2*, *RORA*, *RORC*, and *TIM* was also associated with AHRE burden, which implies that other circadian clock genes may also play an important role in the variability of heart rate change, and then in the development of AF. Further studies are needed to investigate the role of circadian clock genes changes in the occurrence of atrial tachyarrhythmias, including AF.

Macrophage-produced cytokines, including interleukin (IL)-l, IL-6, IL-12, and tumor necrosis factor-α (TNFα), fluctuate in a circadian pattern and generally peak at the end of the rest period. A previous study showed that deleting *BMAL1* or *NRID1* in macrophages enhanced cytokine production and diminished their rhythmic expression [24]. Sleep deprivation has been linked to increased inflammation mediators, including IL-1, IL-6, TNFα, interferon-γ, and vascular endothelial growth factor in humans [25]. These mediators were also reported to be associated with atrial fibrosis and AF episodes [26]. AF could cause atrial tissue structural remodeling and fibrosis, and these were also the hallmarks of AF [27]. Another study reported that the degree of atrial remodeling and fibrosis evaluated by delayed enhancement magnetic resonance imaging could determine and predict AF recurrence after catheter ablation [28]. It is possible that the disruption and dyssynchrony of circadian clock genes expression through sleep disturbance, inflammation, or as a result of disease may lead to AF initiation and perpetuation.

Another interesting finding is that some circadian clock genes expression were slightly increased in patients with paroxysmal AF, and then were significantly decreased in those patients with persistent AF. One plausible explanation is that the increasing circadian clock genes expression may have been a response to AF triggering factors (autonomic system imbalance, inflammation, stress, etc.) or tachyarrhythmia itself, and the response flattened or even depressed under a longer duration of stress and/or tachyarrhythmia. Another possible explanation would be that the different circadian clock genes expression patterns that represent the mechanism of circadian clock genes alterations, resulting in or caused by different AF types (paroxysmal AF or persistent AF), may be different. Further studies on the relationship between circadian clock genes expression and AF type are needed to clarify the underlying mechanism.

Our study also found there was a strong, positive correlation between higher AHRE burden and larger LA size, and that there was also a strong, positive correlation between higher AHRE burden and decreased circadian clock genes expression in patients with AF. Although AHREs are not equivalent to AF and also includes other atrial tachyarrhythmias (atrial tachycardia and atrial flutter), previous studies have shown that a higher AHRE burden was significantly associated with the development of AF [29,30]. The current clinical guideline also recommends using AHREs to inform management for those patients with a previous history of stroke or AF [11]. In the present study, the AHRE burden in those patients with AF already confirmed clinically should be considered as a high representation of the AF burden or at least as an indication of a greater potential of developing AF. Our study showed that a decreased circadian clock genes expression was strongly associated with a high AHRE burden, so it can also be speculated that the depressed circadian clock genes expression may be associated with the occurrence of AF, especially in those patients with a longer AF duration.

Most important of all, the expression of *CRY1*, *NR1D1*, and *RORA*, in addition to LA size, can predict AHRE burden during follow-up. PER and CRY protein accumulate in the cytoplasm and form a heterodimer and translocate to the nucleus to inhibit the transcriptional activity of BMAL1-CLOCK (negative feedback loop). *NR1D1* and *RORA* form the secondary feedback loop and act as repressor and activators of *BMAL1* transcription [4]. Our study results hinted that post-transcriptional regulation of circadian clock genes and the secondary feedback loop may play an important role in the occurrence of AHREs. Further studies investigating the mechanism and effect of these genes and protein expression in the development of atrial tachyarrhythmia are needed.

According to previous studies, LA size is not only associated with AF duration, but is also an independent risk factor for AF and a predictor of recurrence of AF after therapy [31]. With the increasing duration and burden of AF, LA structural remodeling has been observed at both the macroscopic and microscopic level [32]. Our study not only confirmed previous findings, but also uncovered an important finding—that decreased circadian clock genes expression was associated with a higher AHRE burden and a larger LA size. Further studies investigating the mechanism of circadian clock genes disruption related to LA remodeling, AF duration, and AHREs burden are warranted.

There were several limitations in this study. First, we could measure the expression of genes during the daytime only. However, the significant down-expression of several genes at the same time point in persistent AF patients, compared to those with paroxysmal AF or no AF, also implies that there is a link between the disruption of circadian rhythm and persistent AF. Second, other factors, such as diet and light exposure, are already known to influence the expression of circadian clock genes. In the present study, we asked participants to maintain a regular lifestyle before blood sampling, to decrease the influence of these factors on circadian clock genes expression. However, we did not have the survey of well-being, general health (e.g., MOS 36-Item Short-Form Health Survey, EQ-5D questionnaire, Mini-Mental State Examination). Third, this is a study with a smaller sample size event though it already achieved statistical significance. This study may be considered as a pilot study and further larger cohort studies should be performed to confirm the finding. Fourth, the purpose of this study was not to diagnose or predict AF. Therefore, we did not have the sensitivity, specificity, and predictive value of each respective genes. Although our results may not be useful in enhancing the diagnostic ability for AF or AF burden, we would more like to conclude the expression of circadian clock genes are associated with AF type, AHRE burden, and LA size. In addition, the LA size and the expression of some of the circadian clock genes could be used to predict higher AHRE burden in patients with AF. In those AF patients with potential higher AHRE burden, more aggressive ECG monitor and even implantable cardiac device may be indicated to document the AF or AHRE burden and even used for risk stratification. Fifth, we revealed the relationship between the expression of circadian clock genes and different AF types only, and not the causal effects. Further studies investigating the underlying mechanism between circadian clock genes expression and AF are warranted. Finally, we did not analyze the relationship between medication for AF treatment and the expression of circadian clock genes. There are several known regulators of circadian function, such as glucocorticoids, 2-methoxyestradiol, forskolin, PKC and p38 MAPK inhibitors. However, in the current literature to date, no known drugs used to treat AF will affect the expression of circadian clock genes.

## 5. Conclusions

Our study showed that the mRNA expression of circadian clock genes was decreased in patients with persistent AF, compared to those with paroxysmal AF or no AF. The altered expression of circadian clock genes was correlated significantly with a higher AHRE burden and a larger LA size. LA volume and the expression of *CRY1*, *NR1D1*, and *RORA* were significantly associated with AHRE burden. However, the underlying mechanism needs to be elucidated in further studies.

## Figures and Tables

**Table 1:** Baseline characteristics of the 73-subject study population.

Variables	Persistent AF (*n* = 15)	Paroxysmal AF (*n* = 28)	No AF (*n* = 30)	*p*-Value
Age	71.0 ± 8.3	71.0 ± 8.1	72.2 ± 8.7	0.840
Sex (Male/Female)	12/3	12/16 ^a^	9/21 ^a^	0.006
Hypertension	7 (46.7%)	18 (64.3%)	19 (63.3%)	0.481
Diabetes mellitus	6 (40%)	5 (17.9%)	8 (26.7%)	0.287
Previous stroke	3 (20%)	5 (17.9%)	2 (6.7%)	0.338
Heart failure	1 (6.7%)	5 (17.9%)	2 (6.7%)	0.330
Coronary artery disease	3 (20%)	5 (17.9%)	5 (16.7%)	0.963
Chronic kidney disease	3 (20%)	2 (7.1%)	6 (20%)	0.328
Anxiety	4 (26.7%)	8 (28.6%)	7 (23.3%)	0.900
Benzodiazepine	2 (13.3%)	2 (7.1%)	4 (13.3%)	0.713
Non-benzodiazepine	1 (6.7%)	0 (0%)	2 (6.7%)	0.378
Average heart rate	74.5 ± 5.0	73.4 ± 7.9	71.9 ± 5.3	0.488
AHRE burden (IQR)	100 (100–100)	0.5 (0–3.5) ^a^	0 (0–0) ^a^	<0.001
Echocardiographic data				
Left atrium diameter(mm)	49.3 ± 9.3	40.8 ± 10.2 ^a^	38.9 ± 4.4 ^a^	<0.001
Left atrial volume (cm^3^)	102.7 ± 37.5	62.4 ± 43.8 ^a^	50.7 ± 19.2 ^a^	<0.001
Aorta (mm)	32.9 ± 5.1	32.1 ± 4.3	32.7 ± 4.4	0.802
LVEDD (mm)	51.1 ± 8.3	47.4 ± 5.6	48.4 ± 8.3	0.294
LVESD (mm)	35.1 ± 9.4	30.4 ± 4.3	30.8 ± 7.5	0.089
LVEF (%)	60.0 ± 10.9	65.1 ± 7.6	65.9 ± 9.2	0.106
Septal E/e’ ratio	16.3 ± 9.5	13.9 ± 8.9	14.2 ± 9.3	0.740
DT (ms)	181.2 ± 64.6	224.6 ± 72.7	196.7 ± 44.2	0.097
PAP (mmHg)	25.2 ± 10.8	24.9 ± 9.2	24.7 ± 8.4	0.984

Data are expressed as means ± standard deviation, median (interquartile range) or % (n) as an appropriate approach. AHREs = atrial high-rate episodes; AF = atrial fibrillation; DT = deceleration time; E/e’ ratio = the ratio between early mitral inflow velocity and mitral annular early diastolic velocity; IQR = interquartile range; LVEDD = left ventricular end-diastolic dimension; LVEF = left ventricular ejection fraction; LVESD = left ventricular end-systolic dimension; PAP = pulmonary artery pressure. ^a^ *p* < 0.05 versus persistent AF.

**Table 2:** Correlation between burden of atrial high-rate episodes (AHREs) and left atrial size, and correlation between AHRE burden and mRNA expression of circadian clock genes.

Variables	*r*	*p*-Value
Left atrial diameter	0.593	<0.001
Left atrial volume	0.651	<0.001
*BMAL1*	−0.452	0.002
*CK1ε*	−0.244	0.115
*CLOCK*	0.005	0.973
*CRY1*	−0.329	0.031
*CRY2*	−0.516	<0.001
*NR1D1*	−0.518	<0.001
*NR1D2*	−0.410	0.006
*PER1*	−0.216	0.163
*PER2*	−0.473	0.001
*PER3*	−0.306	0.046
*RORA*	−0.508	0.001
*RORB*	−0.424	0.005
*RORC*	−0.569	<0.001
*TIM*	−0.531	<0.001

**Table 3:** Multiple linear regression model predicting burden of atrial high-rate episodes.

Variables	Standardized β Coefficient	*p*-Value
Male sex	0.188	0.066
Age	0.139	0.127
Left atrial volume	0.608	<0.001
*BMAL1*	0.385	0.050
*CRY1*	0.386	0.041
*CRY2*	−0.528	0.382
*NR1D1*	1.149	0.016
*NR1D2*	0.248	0.569
*PER2*	−0.128	0.687
*PER3*	0.148	0.371
*RORA*	−1.676	0.025
*RORB*	0.257	0.112
*RORC*	−0.470	0.064
*TIM*	0.265	0.520

**Table 4:** The burden of atrial high-rate episodes according to tertile distribution on the basis of expression levels of *CRY1*, *NR1D1*, and *RORA* genes.

Variables	Tertile 1	Tertile 2	Tertile 3	*p*-Value for Linear Trend
*CRY1* ^a^	1 (0–27.5)	2.5 (0–97.8)	100 (1–100)	0.047
*NR1D1* ^b^	0 (0–14)	11.1 (0–100)	98.5 (1–100)	0.010
*RORA* ^c^	1 (0–14)	2.5 (0–87.3)	100 (1–100)	0.004

Data are expressed as median (interquartile range). ^a^ Expression level of *CRY1* using the ∆Ct values was classified by tertiles: tertile 1: <9.87, tertile 2: 9.87–10.66, tertile 3: >10.66.; ^b^ Expression level of *NR1D1* using the ∆Ct values was classified by tertiles: tertile 1: <6.11, tertile 2: 6.11–9.00, tertile 3: >9.00; ^c^ Expression level of *RORA* using the ∆Ct values was classified by tertiles: tertile 1: <5.33, tertile 2: 5.33–8.95, tertile 3: >8.95.

## Data Availability

The data presented in this study are available on request from the corresponding author. The data are not publicly available due to privacy.
